# Auditory/visual distance estimation: accuracy and variability

**DOI:** 10.3389/fpsyg.2014.01097

**Published:** 2014-10-07

**Authors:** Paul W. Anderson, Pavel Zahorik

**Affiliations:** ^1^Department of Psychological and Brain Sciences, University of LouisvilleLouisville, KY, USA; ^2^Division of Communicative Disorders, Department of Surgery, School of Medicine, University of LouisvilleLouisville, KY, USA

**Keywords:** spatial hearing, sound localization, distance perception, multimodal, virtual sound

## Abstract

Past research has shown that auditory distance estimation improves when listeners are given the opportunity to see all possible sound sources when compared to no visual input. It has also been established that distance estimation is more accurate in vision than in audition. The present study investigates the degree to which auditory distance estimation is improved when matched with a congruent visual stimulus. Virtual sound sources based on binaural room impulse response (BRIR) measurements made from distances ranging from approximately 0.3 to 9.8 m in a concert hall were used as auditory stimuli. Visual stimuli were photographs taken from the participant's perspective at each distance in the impulse response measurement setup presented on a large HDTV monitor. Participants were asked to estimate egocentric distance to the sound source in each of three conditions: auditory only (A), visual only (V), and congruent auditory/visual stimuli (A+V). Each condition was presented within its own block. Sixty-two participants were tested in order to quantify the response variability inherent in auditory distance perception. Distance estimates from both the V and A+V conditions were found to be considerably more accurate and less variable than estimates from the A condition.

## Introduction

Within the field of human sound localization, the perception of sound source distance has received relatively little scientific study compared to the perception of sound source direction. This is surprising given that the perception of distance is at least as important as direction for conveying important spatial information about our surroundings, such as locating or avoiding auditory objects under conditions when visual information may be ineffective or unavailable. Although generally less is known about auditory distance perception (ADP) than directional perception, it is clear that ADP results in both highly variable judgments (Zahorik et al., 2005) as well as systematic judgment biases (Zahorik, 2002a), especially when compared to directional localization performance, which is comparatively accurate and consistent (Middlebrooks and Green, 1991). In terms of judgment bias, there appears to be general consensuses across a variety of studies and listening conditions that far distances are underestimated while closer distances are overestimated (Zahorik et al., 2005). These results are seemingly at odds with our everyday experience of auditory space that appears to be consistent and relatively accurate. One possible explanation for this discrepancy is that in many everyday situations, ADP may be influenced by additional spatial information provided by other sensory modalities, such as vision. The goal of the current study is to better understand how visual input may influence both bias and variability in ADP.

Visual influences on the apparent direction of a sound source are well-known: The superior spatial resolution of vision dominates, or “captures,” the less precise directional information input through the auditory modality. This effect, which underlies the ventriloquist's illusion, can influence sound sources separated from visual targets by as much as 55° (Thurlow and Jack, 1973). It also appears to be strengthened by temporal synchrony between auditory and visual targets (Jack and Thurlow, 1973), but is unaffected by either attention to the visual distracter or feedback provided to the participant (Vroomen and de Gelder, 2004).

Visual capture also appears to function in the distance dimension. For example, Gardner (1968) demonstrated a form of visual capture, he termed “The Proximity-Image Effect,” in which the nearest visible sound source is mistakenly chosen by listeners to be the actual sound source. Mershon et al. (1980) later discovered that the presence of a visual stimulus does not always elicit an underestimation of the physical distance of a sound source, as Gardner's (1968) data suggest. They found that when an occluded sound source was located closer to listeners than a visible dummy loudspeaker, listeners would overestimate the distance of the sound source as being located at the more distant dummy loudspeaker. Taken together, the results from these two studies clearly demonstrate that the presence of plausible visual targets can influence ADP and that under the appropriate circumstances, this influence results in reduced ADP accuracy.

Under other circumstances, visual information can improve ADP accuracy. For example, Zahorik (2001) demonstrated that ADP accuracy in a reverberant environment improves when listeners have the opportunity to view multiple possible sound sources prior to making judgments. Two groups of listeners were tasked with judging the apparent distance to sound sources along a loudspeaker array. One group was able to view the entire loudspeaker array, and the second group was blindfolded throughout the experiment. Distance judgments provided by the group who were able to view the loudspeaker array were more accurate than judgments from the auditory-only group. Similar conclusions were drawn in a study performed by Calcagno et al. (2012) in which visual cues in the form of LEDs were either present or absent during an ADP task in a dark room. Their setup involved a mobile loudspeaker that was moved along a track between trials and LEDs that were placed at standard intervals along the track. When LEDs were present listeners were informed of the distance to the LEDs prior to the task. Results showed that auditory distance judgments were more accurate when the LEDs were present.

Visual information can also affect the variability of ADP. Results from Zahorik (2001) found ADP variability was reduced in the presence of visual information. However, Calcagno et al. (2012) did not observe a reduction in variability in the presence of visual cues. The reason for these contradictory results may arise from the methodologies used in the two studies. In Zahorik (2001) visual information included information about the room and all possible locations of the loudspeakers. On the other hand, Calcagno et al.'s (2012) listeners were limited in their visual information to LEDs in a dark room. Therefore, more reliable visual distance information in Zahorik (2001) may have led to less variable distance judgments.

Perhaps more interesting are the potential causes of large ADP variability in the absence of visual information. Few studies have explicitly examined this issue given the experimental demands of collecting datasets of sufficient size to reliably quantify ADP variability. Such variability may be conceptualized as originating from at least two sources: one related to the judgments/percepts within a single listener, and one related to differences in judgments/percepts between listeners. Past studies of ADP have not been designed to measure these sources of variability independently. Instead they typically have concentrated on a single source of variability. For example, some ADP studies have utilized a large number (*n* = 80–200) of listeners (Mershon and King, 1975; Mershon and Bowers, 1979; Mershon et al., 1989), but tested relatively few source distances and/or few repetitions per distance. Such designs limit investigation of ADP variability within individual listeners. Other studies (Coleman, 1968; Ashmead et al., 1995; Zahorik, 2002a) have tested greater numbers of source distances with many repetitions at each distance, but at the cost of evaluating fewer individual subjects overall (*n* = 6–9). Zahorik et al. (2005) reanalyzed the results from Zahorik (2002a) to assess ADP judgment variability and found that distance judgments for a sound source may vary between 20 and 60% of the source distance. However, given the relatively small number of listeners evaluated, it is difficult to know how these results may generalize to the population as a whole.

The present study was motivated by gaps in knowledge surrounding the interaction of vision and audition in the distance domain as well as the inherent judgment variability associated with ADP. To assess the degree to which ADP is improved when an auditory stimulus is matched with a congruent visual stimulus, participants judged egocentric distance to a virtual sound source in three conditions: auditory only (A), visual only (V), and congruent auditory/visual stimuli (A+V). Virtual auditory space techniques (Wightman and Kistler, 1989) were used for distance simulation, in order to allow for simple and rapid switching between source distances throughout the experiment. Although based on past results (Zahorik, 2001), it is expected that congruent visual stimuli will result in ADP judgments that are more veridical and less variable, the present study design allows for precise quantification of these variability reduction effects and offers improved generalization to the normal-hearing population as a whole.

## Materials and methods

### Participants

There were a total of 62 (41 female) participants, ranging in age from 18 to 46 (*M* = 22.82). Five participants were removed from analysis: Four because of concerns about their understanding of the task, and due to concerns about self-reported hearing status. All participants had normal hearing based on either self-reports (*n* = 30) or pure-tone audiometric screening (*n* = 32) at 25 dB HL from 250 to 8000 Hz. Informed consent was obtained from all participants prior to data collection, and participants were awarded either monetary compensation or course credit for their participation. All procedures in this study involving human subject participants were approved by the University of Louisville Institutional Review Board (IRB).

### Auditory stimuli

Binaural room impulse responses (BRIRs) were measured from 11 logarithmically-spaced distances ranging from 0.3048 to 9.7536 m at 0° azimuth in a 558-seat concert hall (Margaret Comstock Concert Hall, University of Louisville). The hall had a broadband reverberation time (T_60_) of 1.9 s (ISO-3382, 1997). The auditorium was a complex shape with sloping floors and moveable “clouds” in the ceiling. It had a total volume of approximately 5225 m^3^ (28.956 × 16.9164 × 10.668 m; L × W × H). All BRIR measurements were made with a KEMAR manikin (G.R.A.S. Type 45BM), with IEC711 ear-canal simulators (G.R.A.S. RA0045) and large pinnae (G.R.A.S. KB1060/1) at a fixed location near the edge of the performance stage, facing away from audience seating. The sound source, a high-quality 2-way co-axial loudspeaker (Beyma 8BX) mounted in a sealed 13.5-l cabinet, was moved across the stage to manipulate distance. BRIRs were estimated using Maximum Length Sequence (MLS) system identification techniques (Rife and Vanderkooy, 1989). The MLS signal was 2.73 s in duration (17-th order MLS), sampled at 48 kHz with 24-bit resolution. Five repetitions of this signal were presented and averaged to improve signal-to-noise ratio (SNR), which was <35 dB (0.2–20 kHz) at 9.7536 m.

All BRIR measurements were post-processed to compensate for the response characteristics of the measurement loudspeaker as well as the presentation headphones (Beyerdynamic DR-990 Pro) when coupled to the head. Because residual noise in the measured BRIRs can be easily detectable following virtual sound source synthesis, an additional time-windowing procedure was used to further improve SNR in the BRIRs. The procedure was based on that described by Zahorik (2002a). Briefly, the BRIR was first divided into 30 frequency bands (1/3-octave bandwidth, Gaussian shape) and an energy-decay curve was computed for each band using reverse integration. A straight line was then fit to the decay curve in dB/s over an energy range of −5 to −35 dB. This fit was then used to derive an exponentially-decaying time window for each frequency band. The time windows were then applied in each band, and the results summed across bands. This procedure was effective at improving SNR particularly in the later portions of the BRIR. The source signal for virtual synthesis was a 100 ms sample of Gaussian noise.

### Visual stimuli

**Figure 1:**
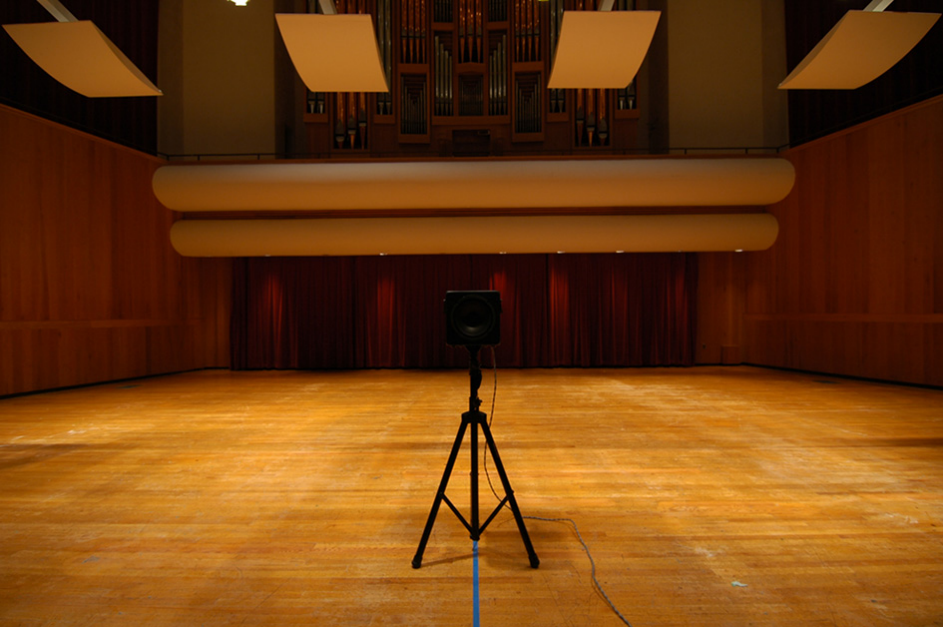
**Visual stimulus example**. A photograph of the measurement loudspeaker was taken at each distance from where the KEMAR mannequin was placed during BRIR measurement at the front of the stage. In the V and A+V conditions a photograph was presented on a large flat screen HDTV and the participant provided a distance judgment to the sound source. In this example, the measurement loudspeaker is placed 2.44 m in front of the camera in Comstock Hall.

Visual stimuli were digital photographs of the measurement loudspeaker taken from the position of the head of the KEMAR manikin (see Figure 1). The camera/lens combination (Nikon D70/Tokina f4 12 mm focal length) produced nearly a 90° field of view. The resulting images (2000 × 3008 pixels) were displayed on a high-quality large screen HDTV (either 46 or 40 in. diagonal). The viewing angle was approximately 51° at the participant's location.

### Procedure

The entire experiment took place in a double-walled sound proof booth (Acoustic Systems, Austin, TX). Participants were asked to estimate egocentric distance to the sound source in each of the three conditions: A, V, and A+V. Participants had the opportunity to play the auditory stimulus multiple times before entering their distance judgment. Once the stimulus was played a distance judgment could be entered at any time. Therefore, some listeners may have only had one exposure to the stimulus on a given trial while other listeners may have had multiple exposures on a given trial (data on the number of times a participant listened to the stimulus were not recorded). In the V and A+V conditions the visual stimulus was present for the entire duration of the trial. Judgments were input using a computer keyboard. Participants had the option of using units of either meters or feet. All judgments were required to be precise to two decimal places, and responses in feet were transformed to meters prior to all data analysis. Listeners were instructed to reserve a response of zero for a percept of inside the head locatedness (Blauert, 1997, p. 132). Most participants (*n* = 45), provided judgments in all three conditions. Each condition was tested within its own block of trials, which included 10 judgments for each of the 11 source distances, for a total of 110 judgments. The order of blocks was counterbalanced, and the order of trials within each block was randomized. An additional set of listeners (*n* = 17), participated only in the A condition and contributed 30 judgments for each of the 11 source distances for a total of 330 judgments. The data from this group of listeners were collected to increase the sample of auditory distance judgments, since we were interested in the amount of intra-subject variability inherent in ADP. Feedback was not provided to the participants. MATLAB software (Mathworks Inc., Natick, MA) was used for stimulus presentation and data collection.

### Data analysis

Following methods used in previous ADP and VDP studies (Da Silva, 1985; Sedgwick, 1986; Zahorik, 2001, 2002a; Zahorik et al., 2005), power functions of the following form were fit (least-squares criterion) to the geometric means in each condition: *ŷ*_*r*_ = *k*Φ^*a*^_*r*_ (*ŷ*_*r*_ = perceived distance, *k* = constant, *a* = power-law exponent, Φ_*r*_ = target source distance). The fit parameters, *k* and *a*, were used as measures of judgment accuracy. The exponent indicates the amount of non-linear compression (*a* < 1) or expansion (*a* > 1) in the function. The constant indicates the amount of linear compression (*k* < 1) or expansion (*k* > 1) in the function. The exponent and constant parameters are equivalent to slope and intercept respectively when perceived distance and physical distance are represented in logarithmic coordinates. Residual error from the fitted functions as well as the proportion of variance accounted for by the fitted function (*R*^2^) were used to describe both between-subject and within-subject response variability. Measures of accuracy and variability were compared between conditions using independent samples *t*-tests with Bonferroni correction. Independent samples *t*-tests were used because not all subjects were tested in all conditions. Intra-subject variability was evaluated using independent *t*-tests comparing listeners in the A condition who performed 10 judgments per distance vs. those who performed 30 judgments per distance. Reliability of distance judgments across conditions was analyzed by computing the Pearson correlations across conditions for the fit parameters and *R*^2^ values. All analyses were performed using MATLAB (Mathworks Inc., Natick, MA), except for the *t*-tests, which were performed using SPSS (IBM Corp., Armonk, NY).

## Results

**Figure 2:**
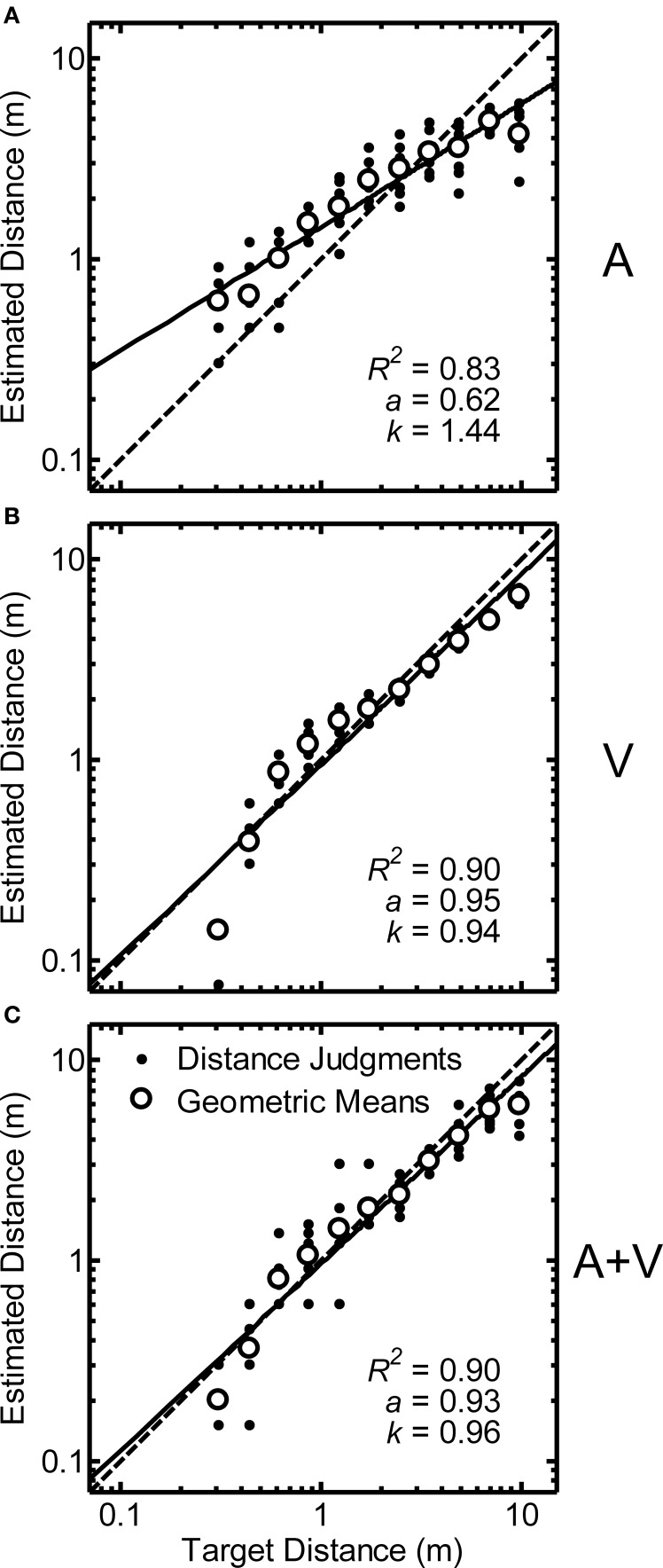
**Data from a single representative participant (code QAD) for auditory (“A,” panel A), visual (“V,” panel B), and auditory/visual (“A+V,” panel C) conditions plotted on logarithmic axes**. Dots show raw distance judgments (*y*): 10 replications/distance. Open circles indicate geometric means (*y*) for each target distance. Data from each condition were fit with a power function (*ŷ*; solid line) of the form *ŷ*_*r*_ = *k*Φ^*a*^_*r*_ (*ŷ*_*r*_ = perceived distance, *k* = constant, *a* = power-law exponent, Φ_*r*_ = target source distance). Fit parameters and the proportion of variability accounted for by the fit (*R*^2^) are shown in each panel. Perfectly accurate performance is indicated by the dotted line in each panel.

Distance estimation results for a single representative participant (Code QAD) are shown in Figures 2A–C for the A, V, and A+V conditions respectively. Dots indicate the raw distance judgments provided by the participant (*y*), while the open circles represent the geometric mean (y) for each distance. The function fits for each condition are plotted as a solid line (*ŷ*), and the diagonal dotted line represents a perfectly accurate relationship between target distance and estimated distance (i.e., *a* = 1, *k* = 1). Each panel includes the fit parameters (*a* and *k*) and proportion of variability accounted for by the fit (*R*^2^). Consistent with previous studies on both auditory (Zahorik et al., 2005) and visual distance estimation (Da Silva, 1985; Sedgwick, 1986), power functions appear to be good fits to the data, although the distance judgments are more accurate and less variable in the conditions with visual stimuli for this participant, as evidenced by the increase in *R*^2^ and the facts that *a* and *k* are closer to 1.

Identical analyses were conducted for all remaining participants in each of the three stimulus conditions. Any distance judgments of “zero” were noted and removed from all subsequent analyses. Of most interest were zero responses in the A condition, since listeners were instructed to only provide a judgment of zero when the stimulus was perceived as located “inside the head.” Only 0.25% of all judgments in the A condition were zero, indicating that the virtual sound sources were perceived as being localized outside the head in the vast majority of cases.

**Figure 3:**
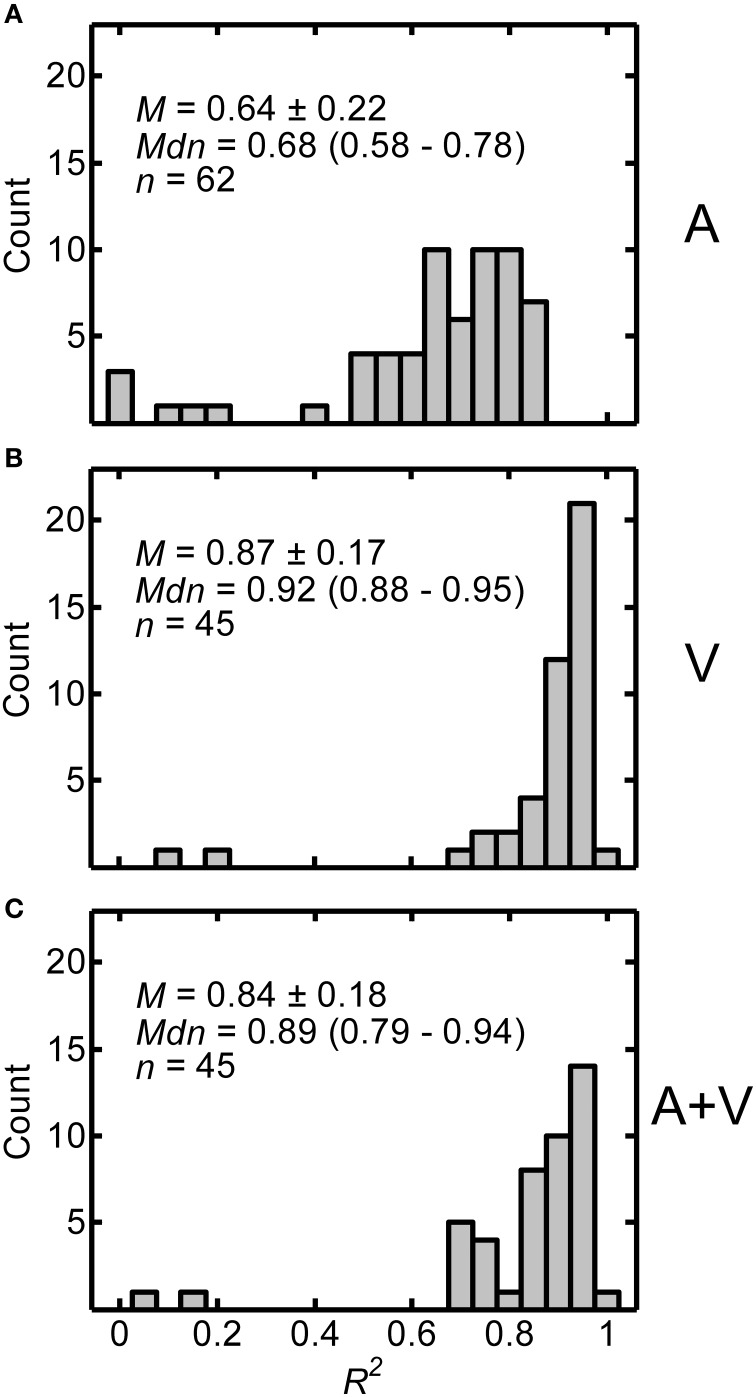
**Distributions of *R*^2^ values from the power function fits for A (A), V (B), and A+V (C) conditions across participants**. Each panel includes the following summary statistics: mean, *M* ± one standard deviation, median, *Mdn* (interquartile range), and number of participants, *n*, in each condition.

The distributions of *R*^2^ values across all participants are displayed in Figures 3A–C for the A, V, and A+V conditions respectively. Because the histograms have a slightly negative skew, both the mean ± one standard deviation and median (interquartile range) are included in each panel along with the number of participants in each condition. High *R*^2^ values indicate that power functions were good fits to the data and support the validity of the calculated power function fit parameters. The *R*^2^ values were generally lower without visual input. The mean *R*^2^ value for the A condition (*M* = 0.638, *SD* = 0.216) was significantly lower than the mean *R*^2^ value for both the V (*M* = 0.874, *SD* = 0.170) and A+V (*M* = 0.836, *SD* = 0.184) conditions, as demonstrated by independent-samples *t*-tests with Bonferroni correction [A vs. V: *t*_(105)_ = −6.085, *p* < 0.0003; A vs. A+V: *t*_(105)_ = −4.979, *p* < 0.0003; V vs. A+V conditions: *t*_(88)_ = 1.012, *p* > 0.945]. Overall, these results suggest that power functions were relatively good fits to the data, but slightly less good for the A condition.

**Figure 4:**
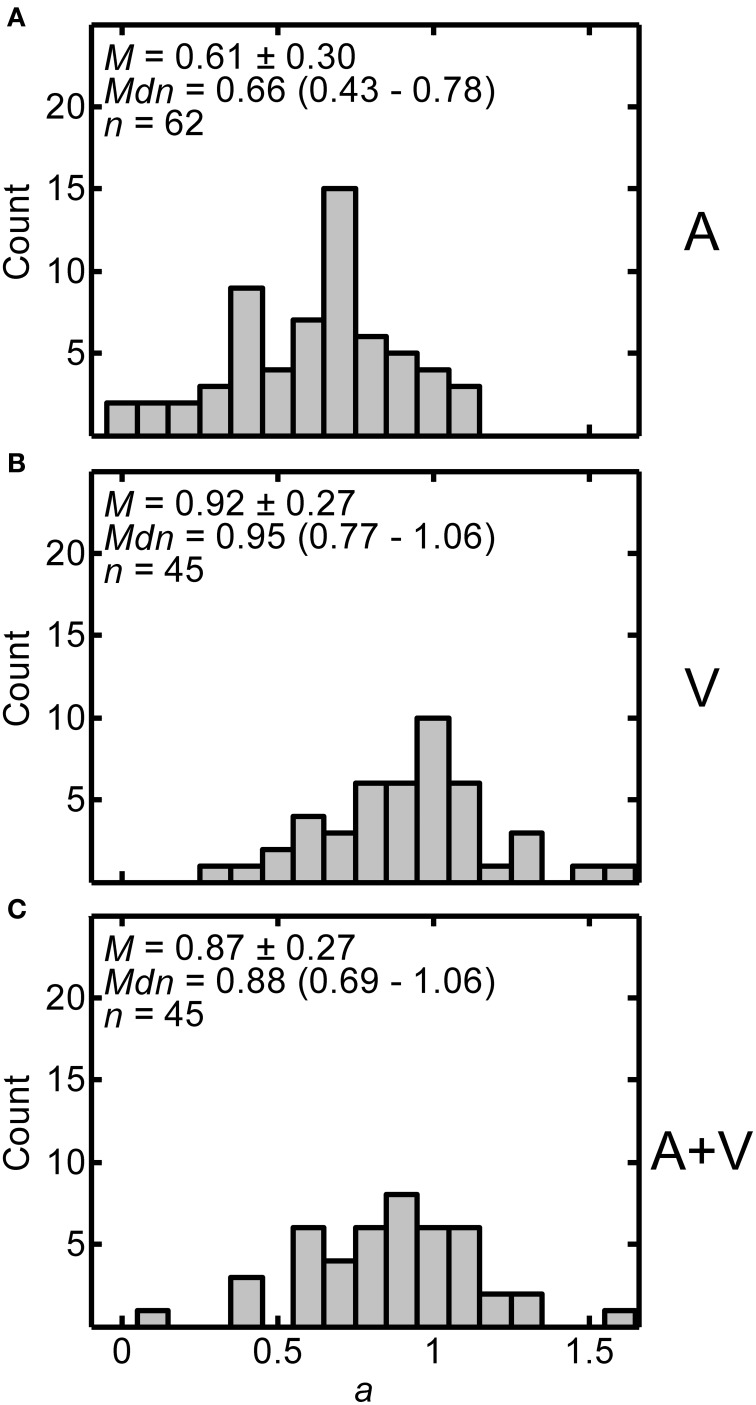
**Distributions of exponents (*a*) from power fits for all participants in A (A), V (B), and A+V (C) conditions**. Each panel includes the following summary statistics: mean, *M* ± one standard deviation, median, *Mdn* (interquartile range), and number of participants, *n*, in each condition.

Exponents from the power function fits provide information about the amount of non-linear compression in the distance judgments. Figures 4A–C display histograms of the exponent values across all participants for the A, V, and A+V conditions respectively. Each panel includes the mean ± one standard deviation, the median (and interquartile range), and the number of participants in each condition. Considerable inter-subject variability may be noted. Using independent-samples *t*-tests with Bonferroni correction, it was determined that the exponents in the A condition (*M* = 0.614, *SD* = 0.299) were significantly lower than the exponents for both the V condition (*M* = 0.916, *SD* = 0.267) and A+V condition (*M* = 0.874, *SD* = 0.271) indicating greater compression in the A condition [A vs. V: *t*_(105)_ = −5.398, *p* < 0.0003; A vs. A+V: *t*_(105)_ = −4.612, *p* < 0.0003; V vs. A+V conditions: *t*_(88)_ = 0.755, *p* > 0.999]. One-sample *t*-tests were also performed to determine whether the exponents in each condition were different from a value of one, which corresponds to no compression. Exponents in all three conditions were significantly less than one [A: *t*_(61)_ = −10.150, *p* < 0.0001; V: *t*_(44)_ = −2.082, *p* < 0.043; A+V: *t*_(44)_ = −3.115, *p* < 0.003], indicating exponential compression in all conditions.

**Figure 5:**
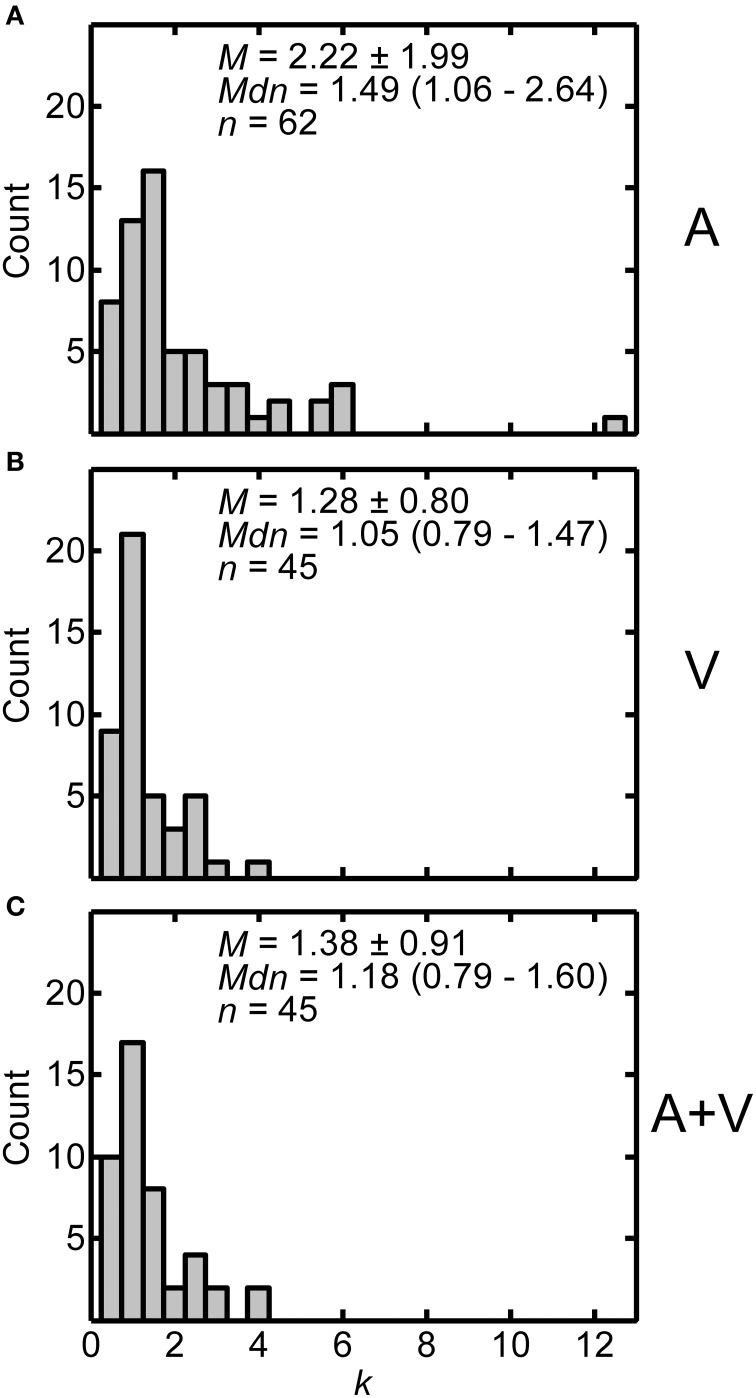
**Distributions of constants (*k*) from power fits for all participants in the A (A), V (B), and A+V (C) conditions**. Each panel includes the following summary statistics: mean, *M* ± one standard deviation, median, *Mdn* (interquartile range), and number of participants, *n*, in each condition.

Constant values from the fits provide information about the amount of linear compression/expansion of the function. Figures 5A–C display histograms of the distributions of constant values across participants in the A, V, and A+V conditions respectively. The histograms are positively skewed, so both the mean ± one standard deviation and median (interquartile range) are included in each panel. Each panel also includes the number of participants in each condition. As in Figure 4, considerable inter-subject variability may be noted. Based on independent *t*-tests with Bonferroni correction, the constants in the A condition (*M* = 2.217, *SD* = 1.992) were significantly greater than constants in either the V (*M* = 1.281, *SD* = 0.801) or A+V conditions (*M* = 1.383, *SD* = 0.912). Overall, these results suggest that near distances are more overestimated in the A condition than in the V or A+V condition. The V and A+V conditions were not significantly different from each other [A vs. V: *t*_(85.359)_ = 3.343, *p* < 0.003; A vs. A+V: *t*_(90.815)_ = 2.904, *p* < 0.015; V vs. A+V: *t*_(88)_ = −0.559, *p* > 0.999]. One-sample *t*-tests confirmed that constants in all three conditions were greater than one [A: *t*_(61)_ = 4.810, *p* < 0.0001; V: *t*_(44)_ = 2.356, *p* < 0.023; A+V: *t*_(44)_ = 2.816, *p* < 0.007], indicating overestimation for distances less than 1 m in all conditions.

**Figure 6:**
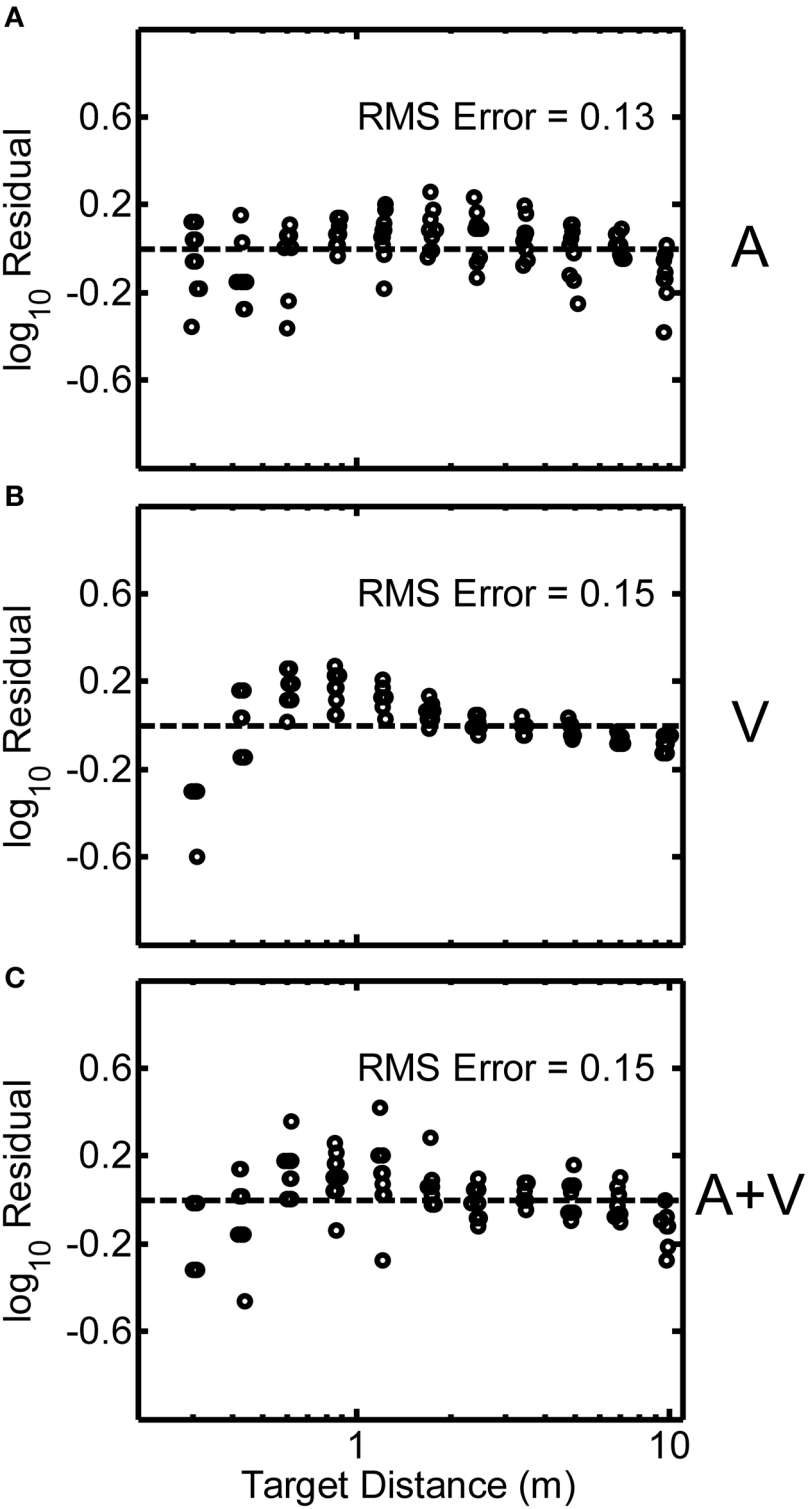
**Log-transformed residuals from the power function fit for a single representative participant (code QAD, see Figure 2) for the A (A), V (B), and A+V (C) conditions**. RMS error across all distances is indicated in each panel. Small random jitter was added to the target distances on the x-axis for visualization purposes.

In order to assess the intra-subject variability of distance judgments, residuals from the power function fits for each participant were analyzed for each condition. Such analyses allow the judgment variability explained by the power function fit to be removed from the data. What remains is an estimate of judgment error independent of the power-law relationship. Figures 6A–C display the log-transformed residuals plotted as a function of target distance in the A, V, and A+V conditions respectively for a representative participant (code QAD, see Figure 2). The RMS error listed in each panel is a measure of average deviation of the responses from the best-fitting power function, and was computed as the square-root of the mean squared deviation of the log-transformed residuals from zero. Although Figure 6B shows the log-transformed residuals decreasing in variability with increasing distance, this pattern is not generally representative of all participants in the study.

**Figure 7:**
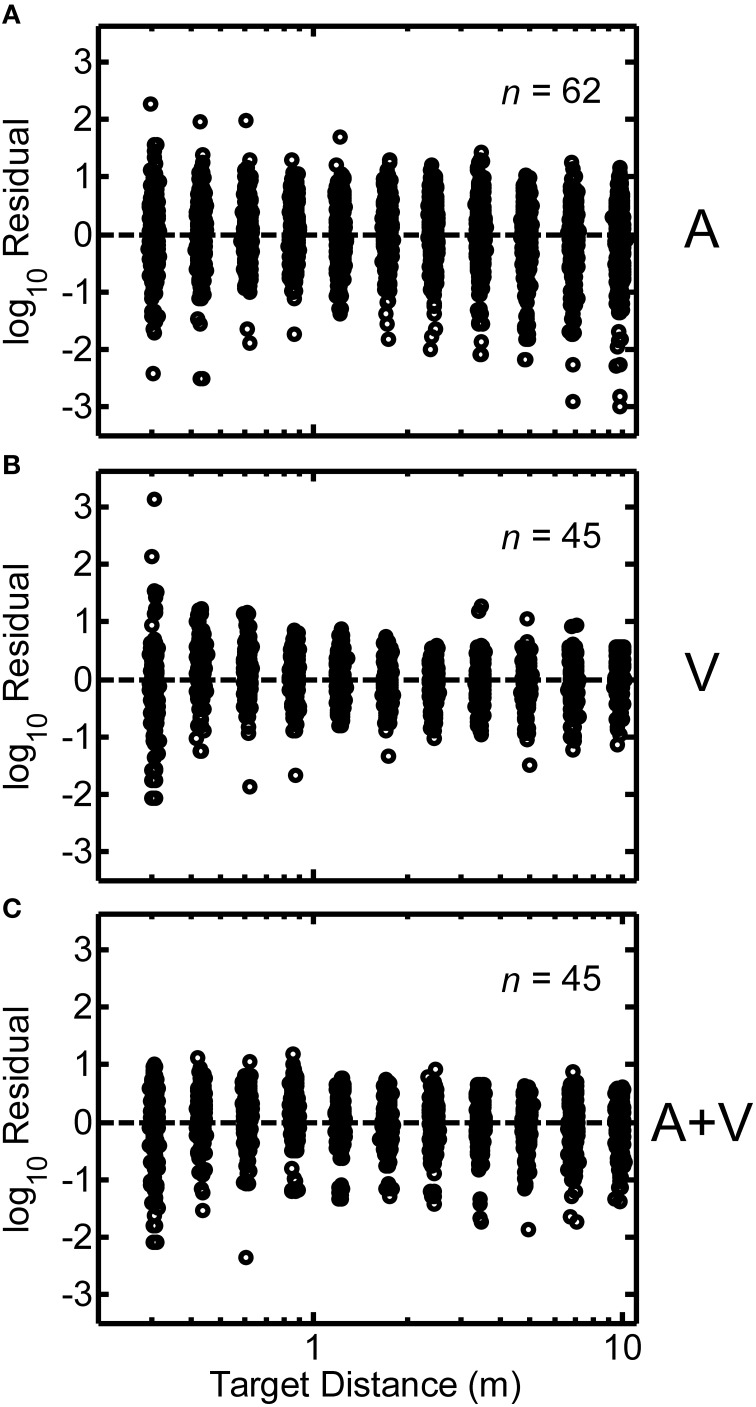
**Same as Figure 6, except results from all participants are shown**. Each panel includes the number of participants per condition. Note that the spread of the residuals is relatively homogeneous as a function of distance.

**Figure 8:**
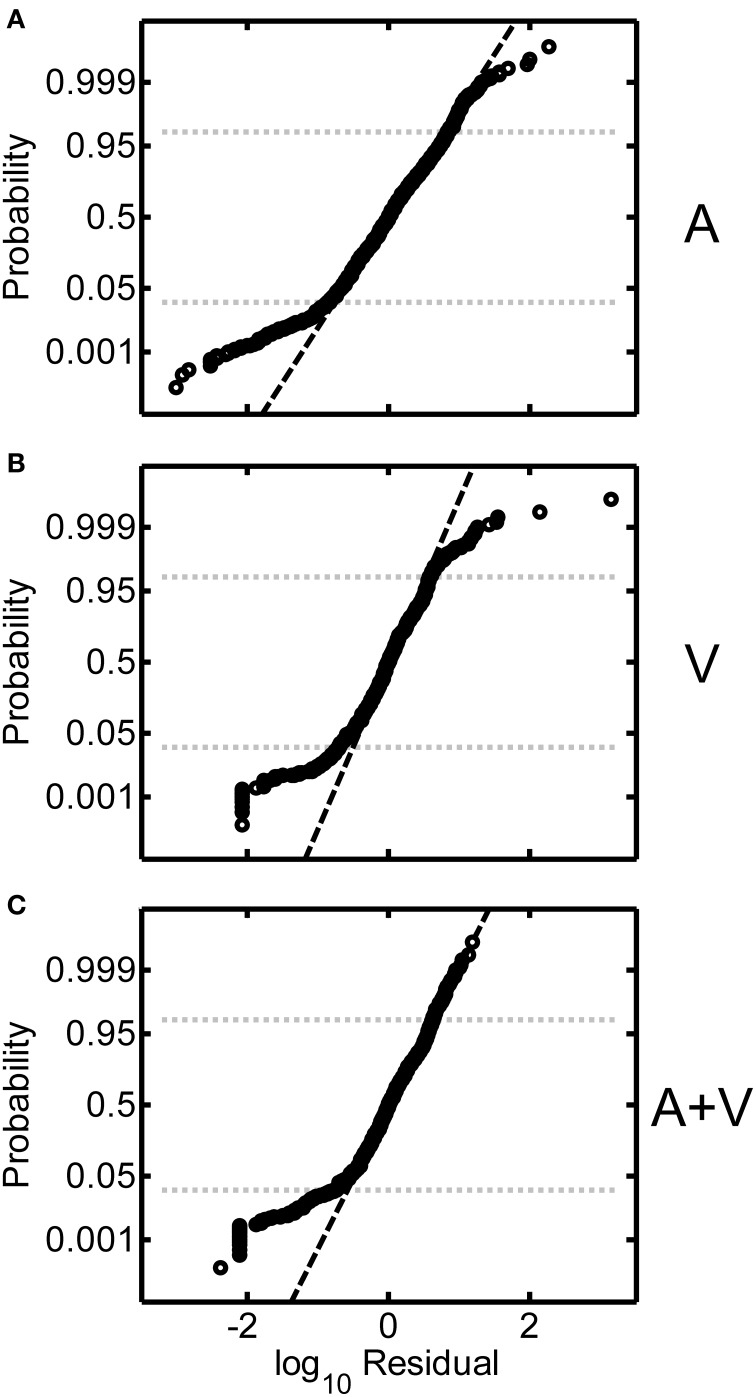
**Normal-probability plots of the log-transformed residuals (all participants) for the A (A), V (B), and A+V (C) conditions**. The dashed diagonal line in each panel indicates normally distributed data. Probability values of 0.025 and 0.975 are shown for reference.

Log-transformed residuals pooled across all participants in the study are shown in Figures 7A–C. These residuals represent error remaining after power functions were fit to the individual subject data. Overall, the spread of the residuals was relatively homogeneous as a function of source distance, which indicates that judgment error was relatively independent of source distance. This was the rationale for our residual RMS error metric, which averages over all source distances. We also examined the distributions of the log-transformed residuals across all target distances. Figures 8A–C display normal-probability plots of the log-transformed residuals collapsed across distance for the A, V, and A+V conditions respectively. The dashed diagonal line in each panel indicates a normal distribution. In all three conditions, it may be observed that the distributions of the log-transformed residuals are very close to normal over a large range of probability values (0.025 and 0.975 are indicated by the dotted lines). Although very extreme values (*p* < 0.025 or *p* > 0.0975) do appear to deviate somewhat from normality, these distribution results are overall consistent with the notion that the underlying internal representation of distance and distance errors are logarithmically spaced (Zahorik, 2002b).

**Figure 9:**
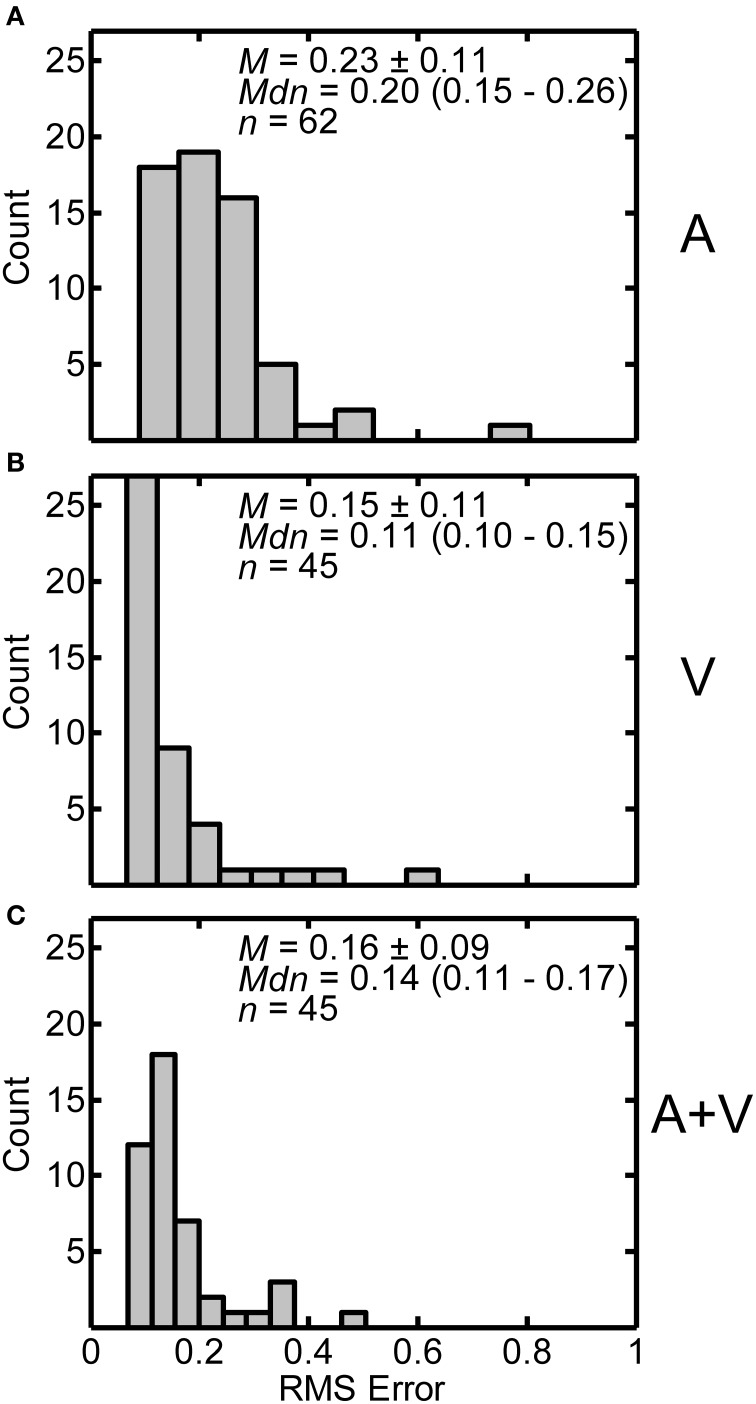
**Distributions of RMS errors from the power function fits from individual participants in the A (A), V (B), and A+V (C) conditions**. Each panel includes the following summary statistics: mean, *M* ± one standard deviation, median, *Mdn* (interquartile range), and number of participants, *n*, in each condition.

Distributions of RMS error in the A, V and A+V conditions are displayed in Figures 9A–C respectively. Each panel includes the following summary statistics: mean ± one standard deviation, median (interquartile range), and number of participants in each condition. The average RMS error for the A (*M* = 0.226, *SD* = 0.111) condition was significantly greater than both the V (*M* = 0.152, *SD* = 0.108) and A+V (*M* = 0.163, *SD* = 0.086) conditions. The V and A+V conditions were not significantly different from each other based on independent samples *t*-tests with Bonferroni correction. [A vs. V: *t*_(105)_ = 3.440, *p* < 0.003; A vs. A+V: *t*_(105)_ = 3.190, *p* < 0.006; V vs. A+V: *t*_(88)_ = −0.523, *p* > 0.999]. These results indicate that when visual stimuli were present, the distance estimates within individual subjects were less variable.

To evaluate the sensitivity of the power function fit procedures to the number of judgments available, fit parameters and *R*^2^ values were compared between participants who performed 10 judgments per distance (*a*: *M* = 0.649, *SD* = 0.259; *k*: *M* = 2.267, *SD* = 2.098; *R*^2^: *M* = 0.650, *SD* = 0.208) and a subset of participants who performed 30 judgments per distance (*a*: *M* = 0.588, *SD* = 0.274; *k*: *M* = 2.130, *SD* = 1.694; *R*^2^: *M* = 0.635, *SD* = 0.201). Independent *t*-tests found no statistically significant difference between the two groups for either fit parameter or *R*^2^ [*a*: *t*_(60)_ = 0.802, *p* > 0.426; *k*: *t*_(60)_ = 0.240, *p* > 0.811; *R*^2^: *t*_(60)_ = 0.246, *p* > 0.806]. These results indicate that 10 judgments per distance is sufficient to reliably estimate the distance psychophysical function.

In order to assess reliability of distance judgments across the three stimulus conditions, correlations between power function fit parameters and statistics were computed. *R*^2^ values in all three conditions were positively correlated [A and V: *r*_(43)_ = 0.660, *p* < 0.001; A and A+V: *r*_(43)_ = 0.674, *p* < 0.001; V and A+V: *r*_(43)_ = 0.922, *p* < 0.001]. This indicates that if a participant's power function fit was good in one condition then it was likely also a good fit in the remaining conditions. Exponents between all three conditions were also significantly positively correlated [A and V: *r*_(43)_ = 0.537, *p* < 0.001; A and A+V: *r*_(43)_ = 0.557, *p* < 0.001; V and A+V: *r*_(43)_ = 0.896, *p* < 0.001]. This indicates that participants with greater amounts of power-function compression, for example, display this trait consistently across stimulus conditions. Similar positive correlations were also observed for the fitted constant values [A and V: *r*_(43)_ = 0.422, *p* < 0.004; A and A+V: *r*_(43)_ = 0.343, *p* < 0.021; V and A+V: *r*_(43)_ = 0.885, *p* < 0.001].

## Discussion

Overall, the results from this study indicate that the presence of visual information improves the accuracy of distance judgments by making the relationship between target distance and judged distance more linear and reducing both inter- and intra-subject variability. These conclusions are based on the results of power function fits to the data in each of the three presentation conditions (A, V, A+V). The decision to fit our data with power functions was based on past reviews of both ADP (Zahorik et al., 2005) and VDP (Da Silva, 1985; Sedgwick, 1986) that used similar methods. Zahorik et al. (2005) fit power functions to 84 datasets from 21 past ADP articles. Da Silva (1985) summarized power function exponents for various visual distance perception studies. Table 1 compares *R*^2^ values and fit parameters (mean ± one standard deviation) from these reviews of past ADP (Zahorik et al., 2005) and VDP studies (Da Silva, 1985), with those from the current study. The summary of VDP exponents only includes studies in which full-cue conditions were used. *R*^2^ values across all conditions and past ADP studies were generally high, which indicates that power function fits were good fits to both past and present data. Exponent and constant parameters from the fitted functions, which provide information about the amount of non-linear and linear compression/expansion of the functions, were, in most cases, similar between past and present studies. The mean exponent from the Zahorik et al. (2005) review was similar (within one standard deviation) to that observed in our A condition. Likewise for the V and A+V conditions, the mean exponents were similar (within one standard deviation) to the mean exponent resulting from Da Silva's (1985) summary. The constant values for the A condition were somewhat higher than reported by Zahorik et al. (2005). Evaluation of these differences is complicated by the fact that the variability of the constant values from the current investigation is much greater. This may be due to variability between subjects in their usage of the response scale that lacked a fixed anchor point. Because the Zahorik et al. (2005) dataset was based on average results from different studies, issues such as this that are related to individual subject variability were minimized, which may have also accounted for the somewhat higher average *R*^2^ values they reported. Despite differences in sources of variability between studies, the fit parameters and *R*^2^ values are all in relative agreement. All are within one standard deviation of each other.

**Table 1:** **Summary of results from past reviews of auditory and visual distance perception studies along with results from the current study**.

**Data source**	**A Condition**	**V Condition**	**A+V Condition**	**(Zahorik et al., 2005)—Audition**	**(Da Silva, 1985)—Vision**
*a*	0.61 ± 0.30	0.92 ± 0.27	0.87 ± 0.27	0.54 ± 0.21	0.99 ± 0.13
*k*	2.22 ± 1.99	1.28 ± 0.80	1.38 ± 0.91	1.32 ± 0.75	
*R*^2^	0.64 ± 0.22	0.87 ± 0.17	0.84 ± 0.18	0.91 ± 0.13	

Power function fit parameters (a and k) and R^2^ (mean ± one standard deviation) are included from each study, except Da Silva (1985) which only provided a summary of exponent, a, values. Results from Zahorik et al. (2005) summarize data from 21 auditory studies. Results from Da Silva (1985) summarize data from 28 vision studies with full depth cues.

Another way to evaluate judgment biases beyond the analysis of the power function fit parameters is to determine the crossover point at which overestimation of close source distances switches to underestimation of farther source distances. This crossover point is the distance at which no bias occurs. Increasing or decreasing either fit parameter moves the crossover point further or closer respectively. Research in vision suggests that the crossover point may be related to a specific distance tendency (SDT; Gogel, 1969), which is the perceived distance of an object reported by participants under conditions with minimal distance cues. Mershon and King (1975) suggested that SDT can also be applied to ADP, given demonstrated tendencies for sounds to be localized toward the crossover point. Specifically, target distances located beyond the crossover point are perceived as closer, and therefore nearer to the crossover point. Conversely sound sources closer than the crossover point are localized farther away, which is again nearer to the crossover point. Mershon and King (1975) also hypothesize that SDT for auditory sources is strongly influenced by the reverberation level of a room. Hence, rooms with similar reverberation characteristics should produce similar SDTs.

In the current study, the crossover point for the A condition was approximately 3.23 m, based on the median exponent and constant parameters from the power function fits. This crossover point is greater than reported by Zahorik et al. (2005) dataset, which was approximately 1.9 m. Because the exponent values were similar in the two studies, it may be concluded that this crossover point discrepancy is caused primarily by the difference in the power function constant parameters. Following Mershon and King's (1975) hypothesis that SDT is related to reverberation level, it seems plausible that these differences in constant values might be linked to differences in the acoustical properties of the rooms used in the two studies. Although the acoustic environments across the data sets analyzed in Zahorik et al. (2005) varied widely, it is likely that the concert hall environment used in the current study had greater amounts of reverberation than the average room in Zahorik et al. (2005) dataset. Greater amounts of reverberation are known to produce greater distance judgments (Mershon and King, 1975), and therefore perhaps greater constant parameters in the power function fits, which in turn produce a more distant SDT. Such conclusions need to be approached cautiously, however, given the large individual variability observed in the constant values, as previously discussed. For VDP, Gogel (1969) found that visual context was necessary to localize visual targets away from the SDT. Reverberation level in ADP may provide the context necessary for sound sources to appear displaced from the SDT.

The observation that distance judgment biases observed in the A+V condition were much lower than the A condition, and nearly identical to those observed in the V condition, we take as evidence of a degree of visual capture in the distance dimension. This result is very similar to the well-known visual capture effects for discrepancies in the angular separation between auditory and visual targets—also known as the “Ventriloquist Effect.” It has been demonstrated that a visual stimulus can bias localization of the auditory sound source when the two are as much as 30° apart in the horizontal plane (Jack and Thurlow, 1973) and 55° in the vertical plane (Thurlow and Jack, 1973). This is a large effect. It is more than an order of magnitude larger than the minimum audible angle that is detectable between two sound sources separated in horizontal angle, which is between 1° and 4° on the median plane (Mills, 1958). Strong visual capture effects have been previously observed in the distance dimension (“The Proximity-Image Effect”) when large discrepancies exist between the auditory and visual targets (Mershon et al., 1980) and particularly when auditory distance information is impoverished (Gardner, 1968). The capture effects observed here are clearly much more subtle.

On the other hand, there are aspects of our results from the A+V condition that are not entirely consistent with visual capture. Research on multisensory perception emphasizes the optimal integration of multisensory information based on the variances of the two modalities (Ernst and Banks, 2002; Alais and Burr, 2004). According to this optimal integration model, the variance of the combined bimodal information should be lower than either modality alone. Additionally, the model stipulates that the modalities are weighted by the inverse of their variance, so the modality with lower variance is more heavily weighted at the modality integration stage of the perceptual process. For example, vision should be heavily weighted in a spatial task; however, if noise is added to the visual stimulus audition will become more heavily weighted. Therefore, if optimal integration occurred in our study, the A+V condition would be expected to have had lower variance than either the A or V condition alone. This was not observed, which is surprising because even if vision in the A+V condition was weighted 100% by the sensory system, the optimal integration theory still predicts lower variance in the A+V condition. It is possible, however, that this apparent lack of optimal integration may relate to the response method used in our study. Magnitude estimation methods are inherently noisier than the discrimination methods used by previous studies that have demonstrated optimal integration (Ernst and Banks, 2002; Alais and Burr, 2004). It is therefore conceivable that the perceptual noise in the A+V condition was in fact lower than either the A or V condition alone, thus consistent with optimal integration, but the response noise was simply too great to observe this reduction in variance consistent with optimal integration. Nevertheless, the measurement of variability is interesting itself because it has not been studied extensively in distance judgment studies.

Finally, our measurements of distance judgment variability provide additional and important insights into ADP and VDP both within and across individual participants. The inherent variability in distance judgments, particularly in the auditory domain, has not been well quantified prior to this study. In general, distance judgment variability across participants was found to be reduced when visual cues were present, a result that is consistent with past work that used similar response and analysis methods for apparent distance judgments (Zahorik, 2001). This result is inconsistent, however, with recent work by Calcagno et al. (2012), which shows essentially constant judgment variability independent of whether visual target information is provided to the listener. This discrepancy could be due to differences in the type of visual information available. In Calcagno's study the visual information (2–4 LEDs in a dark field) was much more limited than the visual information present in either the present study or the Zahorik (2001) study, which provided multiple depth cues to the target locations. It is also worth noting that there were differences in the number of responses evaluated in summarizing response variability (24 judgments/distance in (Calcagno et al., 2012) vs. 959 judgments/distance in this study), as well as the analysis strategies used to summarize variability (variability of raw judgments in Calcagno et al., 2012 vs. variability of log-transformed judgments in this study and in Zahorik, 2001). We also show that when the judgment variability is expressed as logarithmic deviation from a best-fitting power function for individual subjects, the distributions of this deviation (error) measure are approximately normal. This, in conjunction with the fact that power functions are generally good fits to the data, suggests that the perceived auditory/visual space surrounding the subject has a logarithmically spaced topology. This conclusion is consistent with past work related to ADP (Zahorik, 2002b), as well as visual depth work that demonstrates perceptual foreshortening of faraway objects (Wagner, 1985; Loomis et al., 2002).

## Conclusions

Results from this study indicate that: (1) Distance estimates in all conditions (A, V, A+V) were well-explained by power-function fits; (2) The presence of visual targets increased distance judgment accuracy in the V and A+V conditions compared to the A condition; (3) The A condition had greater unexplained response variance than either the V or A+V condition; (4) The unexplained response variance was approximately normally distributed in logarithmic space for all three conditions. These conclusions are consistent with the notion that visual depth information, when available to the participant, dominates the auditory percept of distance. They are also consistent with the idea that aspects of distance perception in both perceived auditory and perceived visual space appear to be organized logarithmically.

### Conflict of interest statement

The authors declare that the research was conducted in the absence of any commercial or financial relationships that could be construed as a potential conflict of interest.
